# Supplementary material for: Three-Station Non-Contrast MR Angiography of the Lower Extremities Using Standard and Centric Fresh Blood Imaging
Source: Sensors (Basel). 2025 Dec 6;25(24):7429. doi: 10.3390/s25247429 (PMC12737181; doi:10.3390/s25247429)
Supplement: Supplementary file 1 [file sensors-25-07429-s001.zip › sensors-3981574-supplementary.pdf]

SUPPLEMENTARY FIGURES

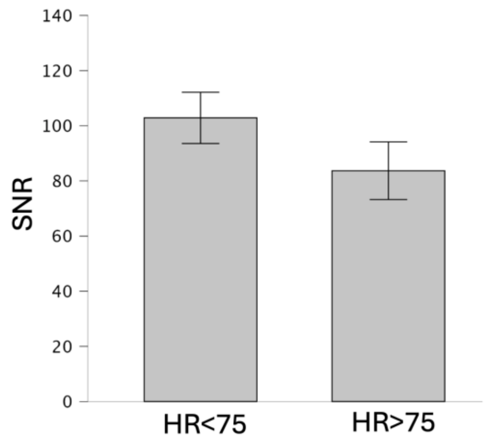

Supplementary Figure S1. Mean SNR values when groups are divided by heart rate (HR) being less than or greater than 75 bpm. Error bars indicate the standard error of the mean.

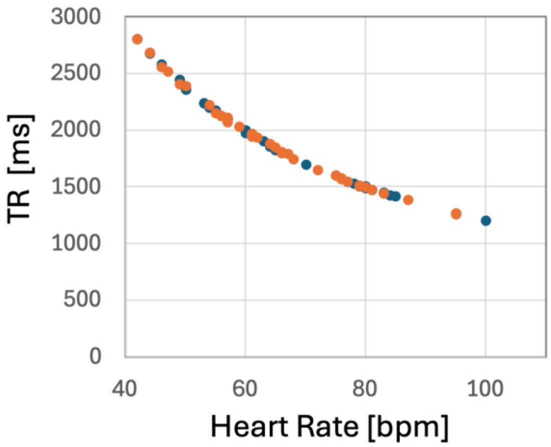

Supplementary Figure S2. Inverse relationship between repetition time (TR) and heart rate. Higher heart rate results in shorter TR and less recovery time, particularly for cFBI.
